# Expression of LMO4 and outcome in pancreatic ductal adenocarcinoma

**DOI:** 10.1038/sj.bjc.6604177

**Published:** 2008-01-29

**Authors:** N C Murphy, C J Scarlett, J G Kench, E Y M Sum, D Segara, E K Colvin, J Susanto, P H Cosman, C-S Lee, E A Musgrove, R L Sutherland, G J Lindeman, S M Henshall, J E Visvader, A V Biankin

**Affiliations:** 1 Cancer Research Program, Garvan Institute of Medical Research, 384 Victoria Street, Darlinghurst, Sydney, New South Wales 2010, Australia; 2 Department of Anatomical Pathology, Royal Prince Alfred Hospital, Missenden Road, Camperdown, Sydney, New South Wales 2050, Australia; 3 Institute of Clinical Pathology and Medical Research, Westmead Hospital, Westmead, New South Wales 2145, Australia; 4 The Walter and Eliza Hall Institute of Medical Research, Parkville, Victoria 3050, Australia; 5 Department of Pathology, University of Sydney, New South Wales 2006, Australia; 6 Division of Surgery, Bankstown Hospital, Eldridge Road, Bankstown, Sydney, New South Wales 2200, Australia

**Keywords:** LMO4, prognosis, outcome, pancreatic cancer, surgical resection, therapeutic response

## Abstract

Identification of a biomarker of prognosis and response to therapy that can be assessed preoperatively would significantly improve overall outcomes for patients with pancreatic cancer. In this study, patients whose tumours exhibited high LMO4 expression had a significant survival advantage following operative resection, whereas the survival of those patients whose tumours had low or no LMO4 expression was not significantly different when resection was compared with operative biopsy alone.

Pancreatic cancer (PC) remains the fourth leading cause of cancer death in western societies with a 5-year survival rate of less than 5%. Pancreatectomy is the only therapeutic intervention that can increase long-term survival. However, only 10–20% of patients who undergo pancreatectomy survive for more than 3 years, and there is no method to predict preoperatively as to which patients will benefit from resection (Yeo *et al*, 1997). For the majority of patients who are unsuitable for operative resection, non-operative approaches, including chemotherapy and chemoradiotherapy, have met with limited success. To date, there are no molecular markers of clinical utility, or rationally designed, molecularly targeted therapies, for PC. Thus, there is a crucial need for the identification of novel molecules important in PC that may also have diagnostic, therapeutic and prognostic utility.

LMO4 is a member of the LIM-only (LMO) family of transcription regulators that act as molecular adaptors, providing a scaffold for multiprotein complexes of DNA-binding factors and transcriptional regulatory proteins (Rabbitts, 1998; Visvader *et al*, 2001; Sum *et al*, 2002, 2005b). Here, we show that aberrant expression of LMO4 occurs in a significant proportion of PCs and that low/no LMO4 expression is associated with a poor outcome in PC. Multivariate analyses identified LMO4 expression as an independent predictor of survival in this cohort and also in a subgroup of patients who underwent pancreatic resection. LMO4 expression cosegregated with resectability and was associated with a significant survival advantage following operative resection. Thus, LMO4 expression may have potential clinical utility in estimating prognosis and response to operative resection.

## MATERIALS AND METHODS

### Patient cohort

We identified a cohort of 120 patients with the diagnosis of pancreatic ductal adenocarcinoma who underwent pancreatic resection or biopsy from Westmead Hospital, Concord Hospital, Royal Prince Alfred Hospital and St Vincent's Hospital in Sydney, Australia (Table 1). This cohort represents a subset of a previously described group of 348 patients (Biankin *et al*, 2002). Multicentre ethical approval for data collection and tissue use was granted by the Human Research Ethics Committees of the above hospitals.

### Immunohistochemistry

Pancreatic tissue microarrays were dewaxed and rehydrated before antigen unmasking, using target retrieval solution (DAKO Corporation, Carpenteria, CA, USA), in a water bath for 30 min. Endogenous peroxidase activity was quenched with 3% hydrogen peroxide in methanol, followed by avidin/biotin and serum-free protein blocks (DAKO Corporation). Rat anti-LMO4 monoclonal antibody was generated as described previously (Sum *et al*, 2005a). Sections were incubated for 30 min in anti-LMO4 monoclonal antibody (20F8) followed by biotinylated rabbit anti-rat IgG (DAKO Corporation). A streptavidin–biotin–peroxidase system was used with 3,3′-diaminobenzidine as a substrate (DAKO Corporation). Counterstaining was performed with Mayer's hematoxylin (DAKO Corporation).

### Immunohistochemical scoring

**Figure 1:**
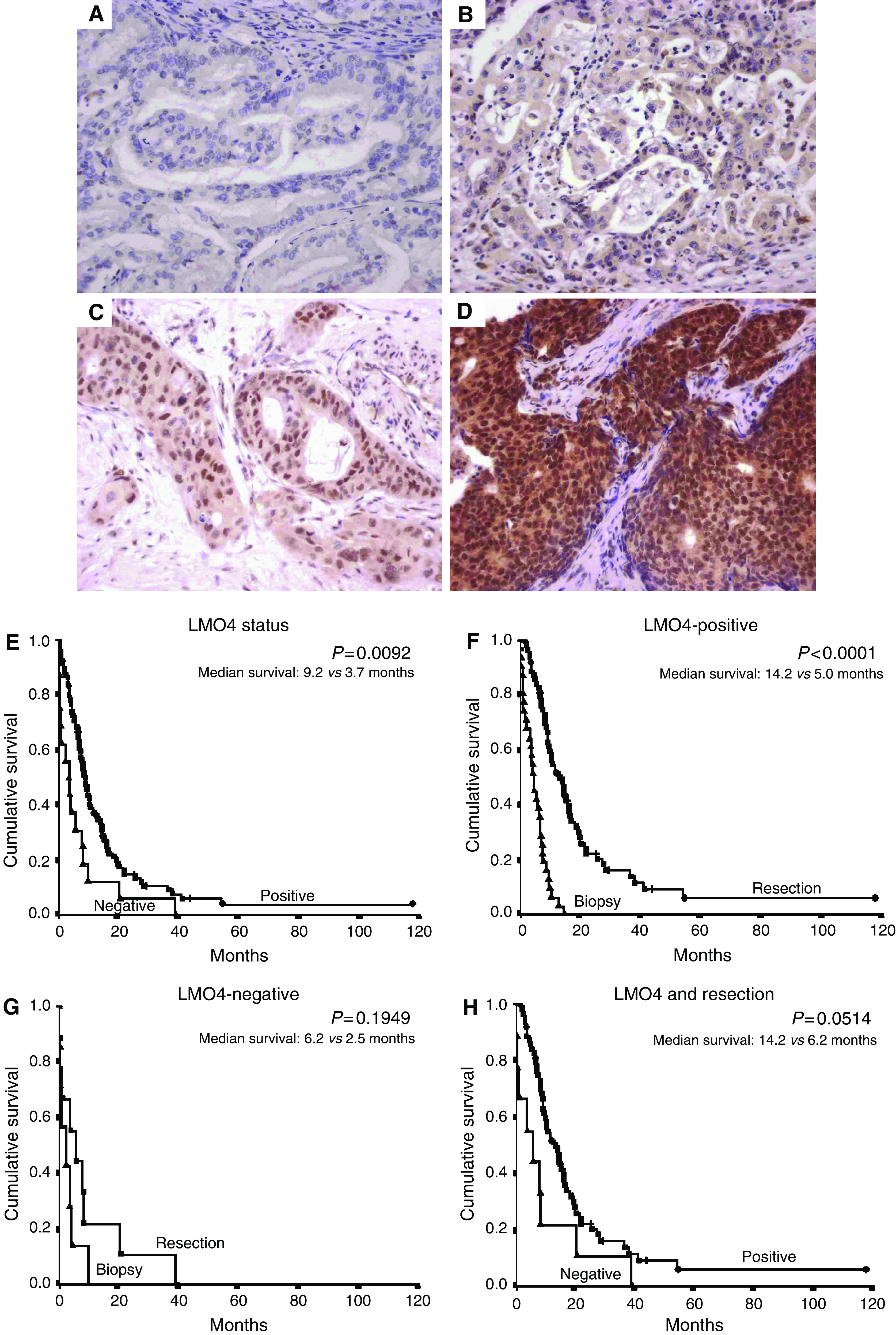
Pancreatic ductal adenocarcinoma: LMO4 nuclear expression score 0–3. (**A**) 0 representing no staining, (**B**) 1 representing mild nuclear staining, (**C**) 2 representing moderate nuclear staining and (**D**) 3 representing strong nuclear staining. Kaplan–Meier survival curves for whole cohort: (**E**) low/no *vs* high (>50% of nuclei with intensity ⩾2) LMO4 nuclear expression. Effect of resection on prognosis in the following subgroups: (**F**) LMO4-positive, (**G**) LMO4-negative; resected cohort: (**H**) low/no *vs* high (>50% of nuclei with intensity ⩾2) LMO4 nuclear expression.

Staining was assessed by two separate observers for each case (DS and JGK), one of whom is a pathologist. Both observers were blinded to patient identification, clinicopathological variables and outcome. Standardisation of scoring was achieved by comparison of scores between observers, and by conferencing, where any discrepancies were resolved by consensus. For resected specimens, an average of 3 × 1.6 mm cores were assessed on tissue microarrays; however, due to limitations in tissue availability, only one or two cores were assessed for biopsy specimens. Scores were given as percentage of cells with positive nuclear staining within the representative area of the tissue microarray core and the absolute intensity of nuclear staining on a scale of 0–3 (0 representing no staining, 1 representing mild nuclear staining, 2 representing moderate nuclear staining and 3 representing strong nuclear staining) (Figure 1A–D). The following criterion was used to achieve a positive score for LMO4 overexpression: nuclear intensity ⩾2 in >50% of nuclei (Sum *et al*, 2005b).

### Statistical evaluation

Statistical evaluation was performed using the Kaplan–Meier survival for univariate analysis and the Cox proportional hazards model for multivariate analysis using the Statview 5.0 software (Abacus Systems, Berkeley, CA, USA).

## RESULTS

### Clinicopathological parameters

Clinicopathological parameters for the cohort are presented in Table 1.

### LMO4 protein expression

Immunohistochemical staining data were analysed in two groups; initially all patients were analysed, then subsequently only the subgroup of patients who underwent pancreatic resections. Significant nuclear LMO4 expression, as defined by the presence of nuclear staining in >50% of tumour cell nuclei with intensity ⩾2, was identified in 100 (83.3%) out of 120 tumours (Table 1).

### Survival analysis

Kaplan–Meier analysis identified that operative resection of the tumours, stage 1 and 2 tumours (lymph node negative) and non-poorly differentiated tumours, was associated with longer survival (Table 1). Overall disease-specific 1-year survival was 32%, with a 3-year survival of 6% and a 5-year survival of 1%.

Low or no LMO4 expression was associated with a poor outcome (Figure 1E). Multivariate analysis using Cox proportional hazards modelling for those factors that were prognostic on univariate analysis identified LMO4 expression status as an independent prognostic factor when modelled together with resection, differentiation and Union Internationale Centre le Cancer (UICC) stage (Table 2, a). This model was refined by stepwise removal of the redundant variable of differentiation (Table 2, b). Operative resection was beneficial to patients who expressed LMO4 (median survival=14.2 *vs* 5.0 months, respectively; log-rank: *P*<0.0001; Figure 1F); in contrast, those patients whose tumours did not express LMO4 had no survival advantage over those who underwent biopsy alone (median survival=6.2 *vs* 5.0 months, respectively; Figure 1G). Hence, in this cohort, LMO4 expression cosegregated with response to operative resection, with patients whose tumours were LMO4-negative having no detectable survival advantage from operative resection.

### Resected cohort

Overall disease-specific 1-year survival following resection was 50%, with a 3-year survival of 16% and a 5-year survival of 6%. Patients in the cohort did not receive chemotherapy as either primary treatment, or in a neoadjuvant or adjuvant setting. Chemotherapy of any type was only given to 10 patients for the palliation of symptoms, and was not associated with a survival advantage.

Survival analysis of patients who underwent operative resection identified a trend toward decreased survival with negative LMO4 expression (borderline statistical significance; Figure 1H). Table 2 (c)–(f) shows the multivariate models for resected cancers. The initial model (Table 2, c) was subjected to stepwise removal of redundant variables to Table 2 (e), where LMO4 expression became the only independent prognostic factor. Removal of margin status demonstrated that LMO4 expression was independent of lymph node involvement (Table 2, f); however, LMO4 expression was not independent of margin involvement by the tumour (Table 2, g).

## DISCUSSION

Low or no LMO4 expression was an independent poor prognostic factor for all patients with PC as well as in the subgroup of patients who underwent pancreatic resection. Importantly, high LMO4 expression was associated with a significant survival advantage following operative resection. In contrast, the survival of those patients whose tumours did not express LMO4 was not significantly different with resection compared with operative biopsy alone. Although operative resection is currently the best method available to treat PC, it is a procedure that carries significant morbidity and mortality. LMO4 status can potentially be assessed using preoperative biopsy material without resection using techniques such as PCR, which overcome the problems of low tissue yield associated with fine-needle aspirate biopsies. Other factors such as tumour size, resection margins, perineural invasion and lymph node status are determined after resection. The ability to reliably predict which patients will or will not benefit from surgery would be a significant advance in the treatment of PC. LMO4 may have a potential role as a marker for determining the suitability for resection and the prognosis of patients with PC.

Deregulation of LMO4 has been previously described in several tumour types including breast, prostate and squamous cell carcinoma of the oral cavity (Visvader *et al*, 2001; Mousses *et al*, 2002; Mizunuma *et al*, 2003). Like the other LMO family members, LMO4 is a transcriptional cofactor that functions as a scaffold for the generation of multiprotein complexes (Visvader *et al*, 2001). Several LMO4-interacting proteins have been identified, including the ubiquitous nuclear adaptor protein Lbd1 (Grutz *et al*, 1998), the transcription factor deformed epidermal autoregulatory factor 1 (Sugihara *et al*, 1998), the basic helix-loop-helix protein HEN1 (Manetopoulos *et al*, 2003) and the grainyhead-like epithelial transactivator (Kudryavtseva *et al*, 2003), as well as the cofactor CtIP and BRCA1.

In conclusion, current prognostic markers in PCs remain poorly defined and cannot be determined preoperatively. Assessment of LMO4 status may provide an additional method for determining the suitability for resection and the prognosis of patients with PC. Although this study demonstrates the potential utility of LMO4 as a prognostic marker for PC, validation of these data is required in large independent cohorts. Further studies to elucidate the pathways regulated by LMO4 and mechanisms by which LMO4 contributes to the development and progression of PC will be an essential step toward further assessing the potential clinical utility of LMO4 expression levels in PC.

## Figures and Tables

**Table 1:** Clinicopathological and outcome data for all patients in the cohort

		**Whole cohort**	***n*=120**		**Resected cohort**	***n*=75**
**Parameter**	**No. (%)**	**Median survival (months)**	***P*-value (log-rank)**	**No. (%)**	**Median survival (months)**	***P*-value (log-rank)**
*Sex*						
Female	50 (41.7)			30 (40)		
Male	70 (58.3)			45 (60)		
						
*Age at diagnosis (years)*						
Mean	64.3				62.3	
Median	66.5				65	
Range	34.4–83.8				34.4–82.6	
						
*Specimen*						
Resection	75 (62.5)	12.2				
Biopsy	39 (32.5)	4.6	<0.0001			
Post-mortem	6 (5)					
						
*Outcome*						
Follow-up		0–117.4			0.2–117.4	
Median follow-up		7.8			10.8	
30-day mortality				2 (2.7)		
Death from PC	106 (88.3)			62 (82.6)		
Death from other cause	2 (1.7)			2 (2.7)		
Alive	8 (6.7)			8 (10.7)		
Lost to follow-up	4 (3.3)			3 (4)		
						
*Stage* a	119					
I	27 (22.7)					
II	10 (8.4)	14.8				
III	67 (56.3)					
IV	15 (12.6)	7.2	0.0001			
						
*Differentiation* b	119					
Well	9 (7.6)			7 (9.3)		
Moderate	65 (54.6)	9.5		44 (58.7)	14.5	
Poor	45 (37.8)	6.2	0.0050	24 (32)	9.7	0.0398
						
*Tumour size*						
⩽20 mm				14 (18.7)	17.1	
>20 mm				61 (81.3)	10.5	0.0455
						
*Margins*						
Clear				40 (53.3)	16.2	
Involved				35 (46.7)	8.6	0.0008
						
*Lymph node status* c				73		
Negative				35 (47.9)	16.2	
Positive				38 (52.1)	9.7	0.0182
						
*LMO4 status*						
Negative	20 (16.7)	3.7		9 (12)	6.2	
Positive	100 (83.3)	9.2	0.0092	66 (88)	14.2	0.0514

PC=pancreatic cancer.

a Stage I and II *vs* stage III and IV tumours for survival analysis.

b Well- and moderately differentiated tumours grouped together for survival analysis.

c Lymph node status was only available in 73 patients in the resected cohort.

**Table 2:** Multivariate analysis for clinicopathological parameters and LMO4 expression in the whole and resected cohorts of PC

**Variable**	**Hazard ratio (95% confidence interval)**	***P*-value**
*(a) Whole cohort (n=120)*		
LMO4 expression	0.535 (0.297–0.961)	0.0364
Operative resection	0.315 (0.191–0.519)	<0.0001
Stage I/II *vs* stage III/IV	1.894 (1.088–3.298)	0.0239
Differentiation	1.252 (0.799–1.962)	0.3275
		
*(b) Whole cohort (n=120)*		
LMO4 expression	0.486 (0.279–0.847)	0.0108
Operative resection	0.313 (0.190–0.515)	<0.0001
Stage I/II *vs* stage III/IV	2.024 (1.182–3.466)	0.0102
		
*(c) Resected subgroup (n=75)*		
LMO4 expression	0.504 (0.229–1.110)	0.0888
Tumour size >20 mm	1.422 (0.697–2.901)	0.3330
Margin involvement	1.575 (0.832–2.981)	0.1629
Lymph node involvement	1.699 (0.912–3.167)	0.0950
Differentiation	1.238 (0.694–2.210)	0.4698
		
*(d) Resected subgroup (n=75)*		
LMO4 expression	0.487 (0.223–1.063)	0.0708
Tumour size >20 mm	1.439 (0.708–2.933)	0.3144
Margin involvement	1.663 (0.892–3.101)	0.1098
Lymph node involvement	1.721 (0.921–3.215)	0.0887
		
*(e) Resected subgroup (n=75)*		
LMO4 expression	0.460 (0.212–0.997)	0.0492
Margin involvement	1.816 (0.990–3.332)	0.0539
Lymph node involvement	1.734 (0.927–3.243)	0.0848
		
*(f) Resected subgroup (n=75)*		
LMO4 expression	0.382 (0.180–0.810)	0.0121
Lymph node involvement	2.215 (1.251–3.922)	0.0063
		
*(g) Resected subgroup (n=75)*		
LMO4 expression	0.579 (0.282–1.191)	0.1376
Margin involvement	0.426 (0.246–0.737)	0.0023

PC=pancreatic cancer.

A and B, multivariate analysis of the whole cohort, with the final model (B) following removal of redundant variables. C–G, multivariate analysis of the resected subgroup. The initial model (C) with stepwise removal of redundant variables through D to E, where LMO4 expression is the only independent prognostic factor. Removal of margin status (F) shows that LMO4 expression is independent of lymph node involvement, but not margin involvement by tumour (G).
